# Touch, feel, heal. The use of hospital green spaces and landscape as sensory-therapeutic gardens: a case study in a university clinic

**DOI:** 10.3389/fpsyg.2023.1201030

**Published:** 2023-11-24

**Authors:** Mihaela Dinu Roman Szabo, Adelina Dumitras, Diana-Maria Mircea, Dana Doroftei, Paul Sestras, Monica Boscaiu, Robert F. Brzuszek, Adriana F. Sestras

**Affiliations:** ^1^Faculty of Forestry and Cadastre, University of Agricultural Sciences and Veterinary Medicine, Cluj-Napoca, Romania; ^2^Institute for Conservation and Improvement of Valencian Agrodiversity (COMAV), Universitat Politècnica de València, Valencia, Spain; ^3^Pharmacy Science and Technology, University of Medicine, Târgu Mureş, Romania; ^4^Faculty of Civil Engineering, Technical University of Cluj-Napoca, Cluj-Napoca, Romania; ^5^Academy of Romanian Scientists, Bucharest, Romania; ^6^Mediterranean Agroforestry Institute (IAM), Universitat Politècnica de València, Valencia, Spain; ^7^Department of Landscape Architecture, Mississippi State University (MSU), Mississippi State, MS, United States

**Keywords:** garden design, healing, hospital green space, sensory garden, therapeutic

## Abstract

It has been documented that patients with mental or physical disabilities can benefit from being placed within the setting of a natural environment. Consequently, the concept of creating spaces that can enhance health preservation or patient recovery, while also augmenting environmental and aesthetic value, has merged as a contemporary discourse. Green areas around hospitals can offer a great opportunity to incorporate healing gardens to benefit their patients and not only. The aim of this paper is to propose a design for a sensory-therapeutic garden based on key principles derived from selected academic literature, focusing on the application of these principles in a healthcare setting in Cluj-Napoca, Romania. The design was informed also by onsite data collection and analysis, and it aims to create a healing landscape that addresses the needs of patients, healthcare providers, and visitors. This study seeks to augment the discourse in the field by demonstrating the practical application of key therapeutic garden design principles in a specific context and how these principles impacted the design process.

## 1 Introduction

Research has shown that human wellbeing and health improves with the ability to spend time outdoors and to have access to green spaces and other forms of Nature (Gidlöf-Gunnarsson and Öhrström, 2007; Bush and Doyon, 2019; Jennings and Bamkole, 2019; de Bell et al., 2020). Our connection to Nature is strongly rooted in our evolutionary process and is an integral part of our regional identity. As humans, we engage with our environment and the natural world on both a physical and psychological level. The physical level refers to the direct interaction between humans and landscape, as a part of a complex, self-regulating system that includes biotic and abiotic elements that work together to maintain and perpetuate the conditions for life on Earth. The psychological level goes beyond what is tangible and delves into the underlying connection existent between humans and their environment (Born et al., 2001; Roös, 2021). This psychological connection is explored in the concept of “biophilia,” which was introduced by Fromm (1973) and later developed by Wilson (1984). The term biophilia refers to the innate attraction humans have toward nature and natural elements and is exemplified in the fields of architecture and urban planning by Kellert and Wilson (1995). The impact of biophilia can be traced to two different origins: first, being in the proximity of nature and visually interacting with natural elements (plants, animals, other people) and second, the response to biomimetic environments, which are designs that incorporate elements inspired by nature (Fromm, 1973; Wilson, 1984; Kellert and Wilson, 1995; Berto and Barbiero, 2017; Salingaros, 2019; Zhong et al., 2022). The mechanisms underlying these responses to the natural environment, as indicated by Philipp (2012), are similar to those of many other complementary and alternative medicine (CAM) therapies. External sensory stimuli releases natural opiates, such as endorphins and enkephalins, in specific regions of the brain. Thus, biophilic environmental stimuli can reduce depressive symptoms, speed up the healing process after stressful situations, and enhance cognitive function (Beute and de Kort, 2018; Gianfredi et al., 2021; Meuwese et al., 2021; Syrbe et al., 2021; Bressane et al., 2022; Szabo et al., 2022; Jin et al., 2023). These scientific insights bring a greater depth to our understanding of the strategies for incorporating biophilia in outdoor spaces design, including in urban design, cities presenting challenges in meeting environmental and societal wellbeing needs. The profound effects of biophilia demonstrate the importance of intentionally designing these spaces with a strong focus on nature, with strategies like integrating natural elements and processes, large variety of plants for a rich sensory experience and biodiversity, create habitats for wildlife, inclusion of water bodies that can add aesthetic value while providing calming sounds, generally use natural materials and mimick natural forms and patterns. A successful biophilic design encourages interaction with the natural world by integrating areas for gardening and sensory experiences, edible landscape, walking trails that incite and create curiosity, while learning about environmental systems, this leading to a stronger connection between man and nature, reinforcing the vital role of biophilia in communities, underscoring the potential for created green spaces to contribute to the environmental, physical and mental health (Soderlund and Newman, 2015; Lin et al., 2018; Xue et al., 2019; Totaforti, 2020; Russo and Andreucci, 2023). These principles find exceptional significance and their potential could be explored especially in spaces used by people that need recovery and care, in the context of healthcare facilities. Green spaces around the hospitals serve as prime locations for fulfilling the potential of biophilic design, forming a key aspect of the built environment, and also playing a significant role in the health and wellbeing of patients (Zhao et al., 2022). This importance transcends active engagement with nature; studies show that, sometimes, even a passive interaction, something as simple as a view of a natural scene from a hospital bed can have positive effects on patients that had surgical intervention, creating feelings of hope and strength that may contribute to a more efficient recovery process (Ulrich, 1986). The healing process consists not only in providing medical care, access to treatment, and medical procedures; but also in providing a healing environment for both physical and spiritual rehabilitation (Bratman et al., 2019; Fancourt et al., 2021), since “healing is a psychological and spiritual concept of health” (Özcan, 2006). The Optimal Healing Environment (OHE) framework suggests that the healing process has both inner and outer implications, and healing spaces that incorporate natural elements may be an important factor that still needs further research (Ananth, 2008).

Looking back in history, ever since the Middle Ages, the outdoor spaces of hospitals were designed in order to provide resting places where the ill could enjoy fresh air. As modern medicine advanced and the architectural design of hospitals became increasingly complex, the incorporation of outdoor healing spaces has been varied in use, new ideas and research directions began to develop, having as goals to explore the therapeutic opportunities of healing gardens or restorative landscapes, bringing together fields like architecture, landscape design and medicine, a blend that begins to reintroduce the ancient connections between healing and nature into the contemporary medical environment (Cooper Marcus and Barnes, 1995; van den Berg, 2005). Gardens, being humanity’s closest connection to the natural environment, have been and continue to be an important adjuvant in the process of stress rehabilitation being not just spaces for recovery but also for interaction and exploration. Furthermore, green spaces can be a statement to the power or adaptability and resilience, their natural cyclicity and transition trough seasons suggest the potential for renewal and growth after periods of dormancy, thus serving as a metaphor for patients on their healing journey, supporting the idea of hope and resilience during challenging times (Nordh et al., 2009; Adevi and Mårtensson, 2013). Current research has shown that gardens can offer sensorial places for autistic children, giving them opportunities to spend time outdoors in a calming environment, or designed to create invigorating landscapes for hyporeactive patients (Barakat et al., 2019; Ghazali et al., 2019). Other conditions, like depression and anxiety are experienced not only by mentally ill patients, but also in cases of convalescence, post-surgery recovery, COVID-19 measures of isolation or prolonged hospital stay. Being involved in outdoor gardening activities can have an ameliorative effect on people suffering from psychological distress by alleviating symptoms (Gerdes et al., 2022; Yang et al., 2022). During the COVID-19 pandemic, the use of green spaces and parks and participating in outdoor activities enhanced young people’s mental health and wellbeing, and was linked to lower the levels of emotional distress (Jackson et al., 2021; Larson et al., 2022). Designing areas surrounding hospitals as therapeutic gardens provides spaces for patients, institution staff, and family members to use and enjoy. It is proven that spending time in the garden walking, contemplating, or engaging in gardening activities could result in a decreased dosage of antipsychotics in dementia affected patients, in mood improvement and increased calmness (Rivasseau-Jonveaux et al., 2012; Whear et al., 2014). This pandemic underscored the critical role that well-designed and maintained therapeutic spaces can play in human health, and one way to ensure the effectiveness of these spaces is by conducting Post-occupancy evaluations.

Post- occupancy evaluation (POE) is a process by which the performance of a built environment is evaluated by users that express the satisfaction level concerning the space. This helps to determine how successful the design intent is and contributes to improving the design of therapeutic spaces (Moore, 1983; Preiser et al., 2015). Even though involving the end-user in the design process helps to create more suitable environments, post-occupancy evaluation is an important tool that offers valuable insight into the newly created space from the perspective of patients, family, and staff (Jiang et al., 2018; Lygum et al., 2019). It might provide information that may not have been considered even during the participatory design process (Buse et al., 2017). The physical environment, even though it has a major influence in a patient’s recovery, is not the only focus a design should have but also the social and interaction aspects, psychological or spiritual implications, thus the exploration and comprehension of the necessities that the final user has, are vital in developing an effective outcome (Abbas and Ghazali, 2011; Ghazali and Abbas, 2012a).

The presence of a garden is highly valued, and participating in garden activities can make nursing home residents feel a sense of familiarity and comfort (Eijkelenboom et al., 2017). Gardening activities or even spending time outdoors in a garden have beneficial implications in the patient’s state of mind, caring for the plants and enjoying the outcome from that process can have therapeutic effects (Piat et al., 2017). Horticulture therapy, along with other forms of conventional therapy can be an adjuvant factor and make a difference in the treatment of individuals suffering from mental disorders. Being involved in activities like fruit harvesting, planting, weeding, provides patients with a sense of purpose, and a valuable opportunity to spend time outdoors (Vujcic et al., 2017).

This paper provides a unique exploration of the opportunities offered by the green spaces around healthcare facilities, specifically, their development as sensory-therapeutic gardens. While similar research focuses on the physical characteristics of such spaces, the present study delves deeper into the importance of user feedback research for the improvement of design strategies for this type of garden. This research integrates findings from a selection of existing Post-occupancy studies conducted in healthcare facilities, underlining how user experiences can meaningfully inform the design process. The gathered information has led to the identification of a set of key principles which were subsequently applied in the design of a sensory-therapeutic garden in a healthcare setting in Cluj-Napoca, Romania. The study bridges the gap between theory and practice, demonstrating how insights from the field can be transformed into design decisions and its contribution lies the way the principles are applied, integrating user feedback, and situational context to enhance the functionality and relevance of therapeutic gardens. This approach can enhance the quality of user’s life while contributing to more sustainable, user-centered healthcare environments.

## 2 Materials and methods

The research methodology for this study consists in three distinct stages. The first stage, was the consultation of various articles focusing on Post-occupancy evaluations of healthcare facilities, with the goal of identifying key principles in therapeutic landscape design. The second stage involved on site visits for data collection and the final stage, the application of these principles, informed by the literature and on site data, in the specific context of a sensory-therapeutic garden design in Cluj-Napoca, Romania.

### 2.1 Literature resources

Although not systematic, a literature exploration was conducted to gather information and better understand the key features of sensory-therapeutic gardens, selecting and examining papers that focus on the results of post-occupancy evaluations of healing spaces. From the examined literature, recurring findings were extracted to form a list of common design principles, emerging from the collective findings of multiple studies and provide a base upon which to construct a therapeutic garden that meets the needs of patients, staff, and visitors. The selected studies, each contributing to these principles, are discussed below.

In the studies by Carnemolla et al. (2021) and Giebel et al. (2022), the primary focus was on the interior design of the building of a residential aged care setting, however, they strongly emphasize the potential role of outdoor environments in enhancing the wellbeing of residents, advocating for the inclusion of natural elements in these facilities, thus providing opportunities for patients, visitors and staff to interact with nature, explore, or simply spend time outdoors. Ryan et al. (2014) and Tekin et al. (2023a,b) note that gardens, as natural healing environments, can offer a welcoming and relaxing space characterized by the presence of vegetation. The type of garden and plants used must be chosen according to the facilities’ context; for instance, an abundance of seasonal plants, even appreciated by most patients, may have adverse effects on cancer patients by constantly reminding them of life’s transient nature; or even traffic noises that can be disturbing. Among the sensory experiences that a garden could offer are: stimulation of the sense of smell using flower fragrance and can be carried by the wind, filling the entire space; and auditory stimulation through moving leaves, rain, birds, and water features. The tactile sense can be stimulated by vegetation through organic textures, by materials such as wood, which visually stand out, giving the space natural features.

Comments left in a visitor’s book in Lady Cilento Children’s Hospital (Australia) revealed that even when located in a crowded area, not having much space, there was still room for a recreational oasis to be built. There was recorded appreciation from the visitors for the numerous seating areas (benches or lawn) to admire the view or colorful plants, or outdoor equipment for physical therapy and secluded spaces for staff recovery (Reeve et al., 2017).

Further design considerations are brought to light in the Whitehouse et al. (2001) study, where, it was observed that a design skewed toward hardscape elements, led to suggestions from users for more diverse vegetation, including shade-providing trees. Younger users expressed a desire for more activities within the garden, such as gardening tasks and plant identification opportunities. Existing garden features such as the presence of water and interactive elements, the plants decor, the bright colors, and the opportunity it provides to spend time outdoors were elements mentioned as relevant and helpful in the design. These examples illustrate the complexity of users needs and preferences in the context of sensory-therapeutic garden design, and further inform the evolving list of design principles.

A POE study of a rooftop hospital garden by Davis (2011) concluded that providing easy access and visibility to the garden encourages exploration. The study mentioned that the space should be organized to avoid ambiguity and help patients get around instead of creating confusion, and long-term maintenance should be another aspect to consider in the design process. Providing comfortable shaded seating is recommended, and also creating secluded spots for individuals who want privacy might contribute to the design’s use. This understanding is reinforced by research conducted across various children’s hospitals in Texas, where the provision of shade was found to create a desirable ambiance and positively influence visitor decisions to spend time in the garden (Pasha, 2013). The quality and quantity of seating areas also directly impacted the frequency and duration of visits. Children, being more active, particularly benefited from a diverse range of garden activities. Additionally, staff members were found to value secluded areas where they could take breaks away from patients, walk or socialize. Notably, staff visits to the garden were seldom solitary, indicating a demand for personal space and time (Sherman et al., 2005; Naderi and Shin, 2008).

The role of gardens extends beyond the mere provision of a physical space. The literature points to a profound psychological impact resulting from the presence and use of a garden (Boffi et al., 2022). For example, Eijkelenboom et al. (2017) suggests that the simple presence of a garden in nursing homes settings greatly contributes to residents’ confort and familiarity, furthermore, the act of getting involved in gardening activities has been proven to have therapeutic benefits, improving the mental state of patients (Piat et al., 2017). Horticulture therapy, along with other forms of conventional therapy can be an adjuvant factor and make a difference in the treatment of individuals suffering from mental disorders. Being involved in activities like fruit harvesting, planting, weeding, provides patients with a sense of purpose, and a valuable opportunity to spend time outdoors (Davis, 2001; Vujcic et al., 2017). These findings underline the importance of not only incorporating a garden within the healthcare facility but also creating opportunities for users to interact with the garden, further informing the principles for designing sensory-therapeutic gardens.

Based upon the findings in the selected literature, a series of recurring design principles have been identified, supporting the creation of sensory-therapeutic gardens that meets the needs of patients, staff and visitors:

a. Contextual design: the selected vegetation and the design of the space should fit the specific context of the healthcare facility, factors like climate, native species and specific user demographics should guide design decisions.
b. Organization: the design should minimize ambiguity and confusion risk, be well organized, facilitating user navigation through the space.
c. Accessibility and visibility: the garden should be visible and inviting, easily accessible to all users.
d. Sensory stimulation: the design should leverage vegetation and natural processes for sensory stimulation.
e. Biodiversity: the presence of wildlife, such as bees, butterflies, squirrels should be encouraged.
f. Shade and seating: in order to meet users’ needs for relaxation and rest, plenty of seating areas and shade should be provided.
g. Softscape dominance: a dominance of vegetation over the hardscape elements is more appreciated.
h. Water features: including water elements in the design can improve users’ sensory experience and therapeutic qualities of the space.
i. Staff privacy: private areas for staff are necessary to be included in the design.
j. User interaction with nature: opportunities for the user to interact with the garden and natural elements directly should be provided through activities (planting, weeding, fruit/medicial plants harvesting, sensory experiences).

### 2.2 The design process

The design process began with the identification of design principles in literature exploration, which later guided the design, translating them into the unique context of the site in Cluj-Napoca, Romania.

**FIGURE 1:**
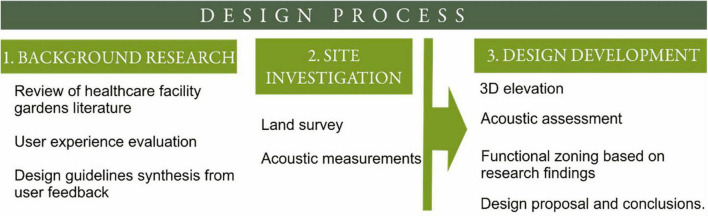
Design process.

The second stage consisted in an onsite visit for data collection (sound measurements using Decibel Meter and Recorder eS528L, ennoLogic), developing a comprehensive understanding of the site’s topography, and in order to create a visual record of the site’s current state, photographic documentation was carried out. These specific site conditions, coupled with the insights from the literature, informed the design decisions, ensuring that the resultant design was both theoretically sound and contextually appropriate. The last stage consisted in applying the insights from the design principles to incorporate them into the sensory-therapeutic garden design concept, in order to create a space for healing and wellbeing. In order to validate the garden design we analyzed how well the key features revealed by the scientific literature were integrated into the design concept (Figure 1).

For the representation of graphical elements such as 2D plans or renderings, CorelDraw Graphics Suite X8, SketchUp Pro 2021 and Photoshop CS5 software were used.

### 2.3 Design proposal and representation of the graphical elements

#### 2.3.1 Site location and context

**FIGURE 2:**
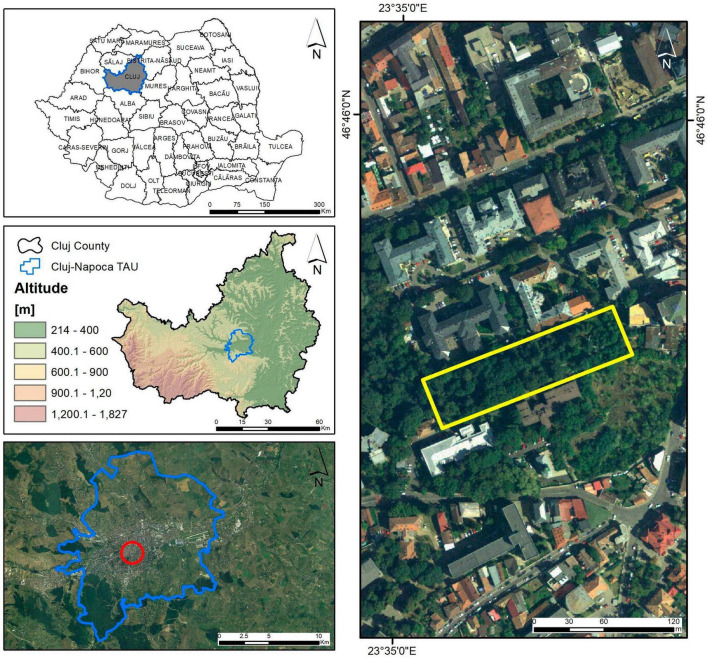
Geographic location of the study area; TAU: territorial administrative unit.

**FIGURE 3:**
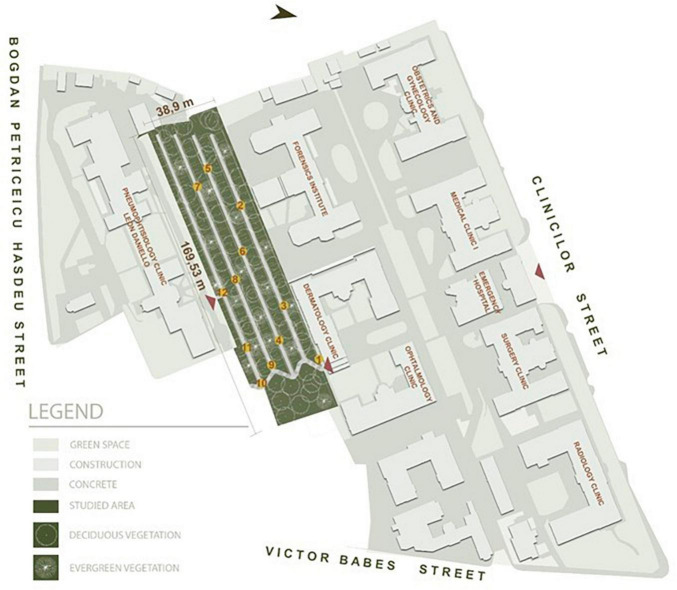
Site location.

The studied site is located in Cluj-Napoca, an academic city in the North-Western part of Romania (Figure 2). The site is close to the city center, being part of the Universitary Clinics Ensemble. It is represented by a terraced green space, having a 6,593 m^2^ surface in the near proximity to the Ophthalmology, Dermatology, Pneumophtisiology, and Forensic Clinics and close to the Emergency, Radiology, Gynecology and Surgery Clinics (Figure 3). In the early plans of the Clinics, this green space was built to provide recreational space for the patients and the medical staff. Nowadays, the area is used only for transit due to its degradation.

#### 2.3.2 The studied area—Current situation

**FIGURE 4:**
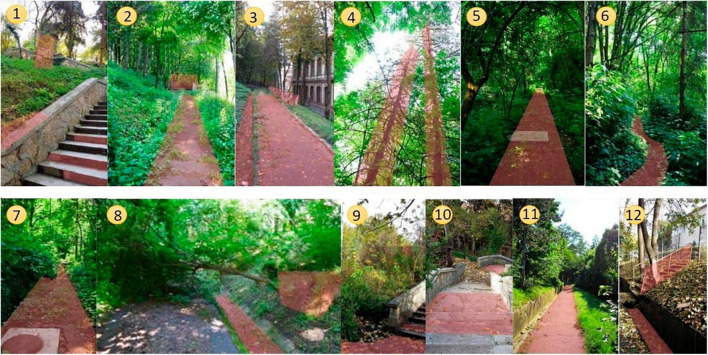
Site photographies.

As the photographs taken on the site show (Figure 4), the terrain has abundant natural vegetation invading the space and overshadowing it (*Fraxinus excelsior*, *Acer pseudoplatanus*, *Rosa canina*, *Rubus idaeus*, *Sambucus nigra*, *Fagus sylvatica*, *Phalaris arundinacea*, *Campanula rapunculoides*, *Hedera helix*), sometimes with fallen trees blocking the way. The drainage system is clogged by vegetation and the pathways are also covered with obstruction by plants. The concrete railing, the stairs, and the alleys show significant signs of decay (Figure 4).

#### 2.3.3 Terrain morphology

**FIGURE 5:**
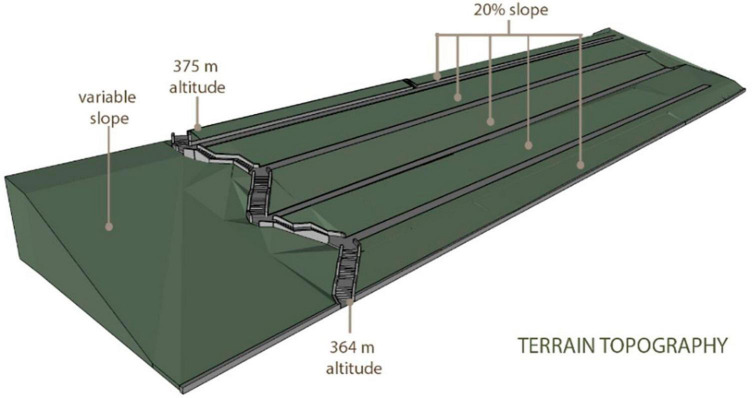
Terrain topography.

The studied site consists of four terraces and five slopes of approximately 20% and the 11 meters difference between the areas is linked by concrete stairs. At the edges of the terraces, the land is unlevel. The terrace in the proximity of the stairs has a variable slope, and one can observe that in the past, that zone was also terraced (Figure 5).

## 3 Results

### 3.1 Noise pollution study

**FIGURE 6:**
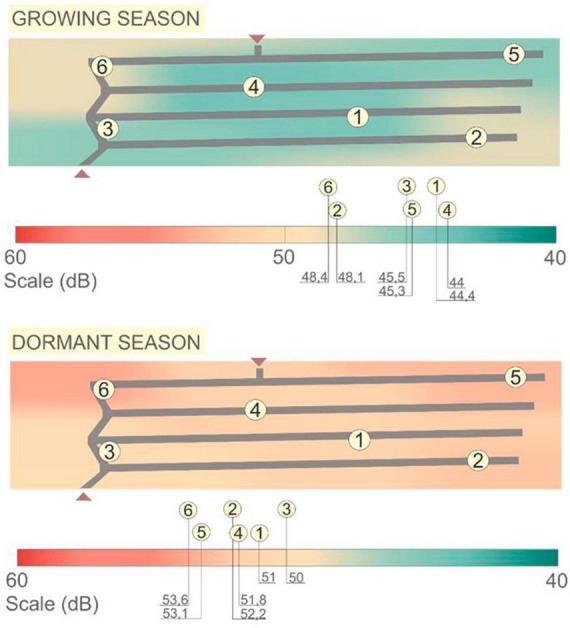
Noise pollution study.

Even though the site is located far from the surrounding streets, a noise pollution study was needed to reduce noise levels. The human ear is comfortable with sounds between 40 and 60 dB, and the site resides in this range both in the growing season and in the dormant period, with higher measured levels of noise during the leafless stage. The central areas are more protected by vegetation during the growing season while the peripheral zones are a bit affected by nearby traffic (Figure 6).

### 3.2 Design proposal—Zoning and masterplan

A major design recommendation would be for the space to be cleaned of unwanted vegetation, keeping only the healthy trees providing shade—a key feature identified in the literature review. Additionally, it’s advisable to trim the overcrowding indigenous bushes so that the sun and light make room inside the space.

**FIGURE 7:**
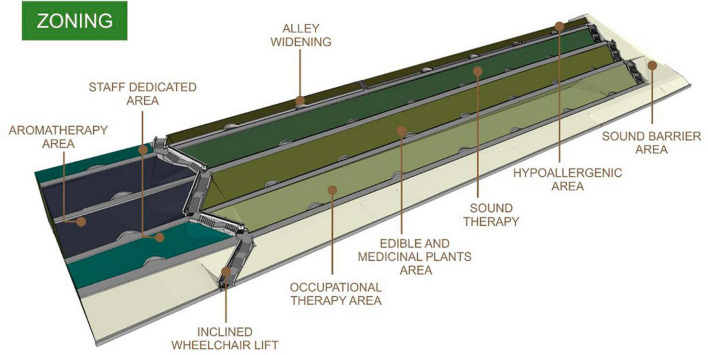
Zoning.

Based on the design principles and the results of the site visit, the proposed design has a primary aim to create a recreational space that alleviates the patient’s state, acts as adjuvant in the healing process and encourages the visitor’s interaction with nature, by using diverse textured plants or materials, and create activity-engaging opportunities like fruit or flower harvesting, gardening or long walks. This approach addresses the principle for providing opportunities for users to interact with the garden directly. Due to the present configuration of the terrain, it is considered opportune to split the site into seven main areas. Each terrace-slope ensemble would have specific tasks creating platforms with different purposes (occupational therapy, edible and medicinal plants, sound therapy, hypo-allergenic area). The area near the stairs would serve two purposes, the central part would be dedicated to an aromatherapy area. The upper and lower extremities, being isolated, would create spaces for the medical staff to relax, supporting the principle for a more private area for staff. The peripheral areas would act as sound barriers, for the possible much-elevated noise values after the unnecessary vegetation removal (Figure 7).

**FIGURE 8:**
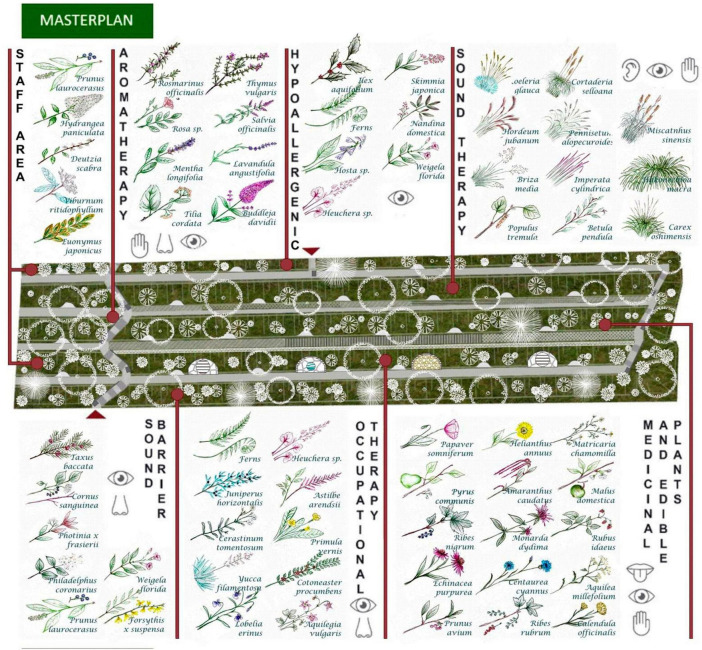
Masterplan.

It is considered that the purposes of the created areas would serve not only the patients with sensory deficiencies (ophthalmological or dermatological) but would also act as comfort zones for post-surgery recovering patients, new mothers, and individuals with respiratory conditions. This fits the clinics’ context, as one of the key features suggested, while also providing outdoor space for visiting family or medical staff. The occupational therapy, aromatherapy, edible and medicinal plants areas are more activity oriented, and the rest of the garden focuses more on nature contemplation and relaxation, bringing balance to the static/dynamic ratio of the garden. Every zone would have plenty of seating areas regardless of its use, no matter if they would be used by the patients or companions. Given the fact that the stairs are the main and only access in the garden, installing an inclined platform stair lift for disabled people would be crucial. This will facilitate access as the literature design principles suggest, and additionally create widened spaces in the alleys at 15-meter intervals for the wheelchairs to comfortably pass each other, as the alleys maximum width does not pass 1.70 meters (Figure 7). The area would be accessed through the four long alleys that cross it from one end to the other in order to prevent confusion in perceiving the environment. Also, the entrance in each zone would have signage so that the visitor would have a choice whether to explore that area and create expectations concerning the possible experiences, and provide visibility and clarity to the space, according to the principles (Figure 8). Since the presence of wildlife is a key feature in a healing garden design, animating the spaces and enchanting the viewers, the installation of bird houses and feeders, insect hotels and bat boxes is recommended in each area. Overall, the proposed design primarily seeks to create a recreational space that aids in the healing process, promotes interactions with nature and provides engagement opportunities, meeting the principles of a well-organized space, being aligned with the facility profile, providing direct access and visibility.

### 3.3 Aromatherapy area

The space near the stairs would be an extension of the existing terraces, thus creating continuity in the design and accessibility. The central area of this space would be dedicated to aromatherapy. Besides the olfactory stimulating vegetation and resting places, bird baths and container ponds would bring movement and sound, by the presence of water elements, encouraging wildlife, offering sensory stimulation and integrating water features being between the found key features. Plant harvesting would be encouraged and have not only a momentary effect, but allow patients to keep near their beds the flowers they have gathered would extend the pleasant olfactory experience they had outdoors. This would offer opportunities for the patients to interact with the garden directly, as the design principles suggest. The proposed plant material that would reflect and enhance the functions of this area, along with various features, are described in Table 1. The profile of the area would be designed accordingly to the facility profile, contributing to the overall organization of the space having a central location and accessibility, and meeting other literature key features like shaded sitting areas and softscape dominance.

**TABLE 1:** The proposed plant material that would reflect and enhance the functions of the intended area, along with various features.

Species	Aromatic properties	Therapeutic effects	Decorative elements	Tactile experience	Plant type/growth habit/decorative period
*Tilia cordata*	Soft pleasant scent; attracts pollinators	Contains volatile organic compounds that released into the air have calming effect; its scent can help promote relaxation and create a peaceful atmosphere (Arcos et al., 2006; Al-Essa et al., 2007; Redzic, 2010)	Heart-shaped vibrant green colored leaves; clusters of cream-colored flowers	Rough mature bark, smoother texture of younger trees or branches; delicate flowers, soft leaves	Perennial/tree/early to mid-summer
*Buddleja davidii*	Sweet, honey-like fragrance; attracts pollinators	Mood improvement; positive effects on the respiratory and nervous systems (Hikal et al., 2022)	Colorful inflorescences; soft, grayish-green foliage	Rough bark on the stems and branches; smooth fuzzy textured leaves; soft, velvety flowers	Perennial/shrub/summer-early autumn
*Lavandula angustifolia*	Sweet, floral, herbaceous scent; attracts pollinators	Calming effects of the nervous system; improves sleep quality and alleviates headaches; reduces stress, anxiety, and symptoms of depression (Yaghoobi et al., 2016; de Melo Alves Silva et al., 2023)	Gray-green foliage; dense spikes or purple flowers	Soft textured leaves; it releases a gentle fragrance when the leaves or flowers are rubbed, adding to the sensory experience; soft, delicate flowers with velvet texture	Perennial/shrub/late spring to late summer
*Mentha longifolia*	Fresh scent, slightly sweet; attracts pollinators	Cooling and invigorating effect on the senses, stimulates the mind and improves concentration (Hanafy et al., 2020)	Small lilac flowers arranged in whorls; bright green colored leaves, grow densely on the stems, creating a lush appearance	Soft slightly velvety textured leaves; square-shaped slightly ridged stems; it releases a gentle fragrance when the leaves are crushed, adding to the sensory experience	Perennial/herbaceous/mid-summer-early autumn
*Salvia officinalis*	Strong, herbaceous, slightly floral, earthly scent; attracts pollinators	Reduces stress and anxiety, enhances mental clarity, and improves memory (Kennedy et al., 2005)	Gray-green leaves; small tubular purple flowers that grow in whorls along the stems	Leaves and stems with slightly rough texture	Perennial/herbaceous/late spring to early summer
*Rosa damascena*	Rich, floral scent; attracts pollinators	Mood-enhancing properties; petals can be used in herbal tea blends or as natural air freshener (Vinokur et al., 2006; Baydar and Baydar, 2013)	Abundant pink flowers; pinnate green leaves	Soft, velvety petals	perennial/shrub/late spring to early summer
*Rosmarinus officinalis*	Strong, woody, refreshing scent; attracts pollinators	Improves mental clarity, focus and memory; has calming effect on the nervous system, helps relieve stress and anxiety; used in aromatherapy to ease respiratory problems such as congestion and coughing (Hamidpour et al., 2017; Rahbardar and Hosseinzadeh, 2020)	Needle-like leaves, dark green and glossy; small, blue-violet flowers	Slightly rough leaves with a waxy surface; It releases a gentle fragrance when the leaves are crushed, adding to the sensory experience	Perennial/shrub/early spring to late summer
*Thymus vulgaris*	Strong pleasant, herbal aroma with hints of mint and slightly spicy; attracts pollinators	Supports respiratory health; has calming and soothing scent; improves focus and concentration (Rizwan, 2021)	Small narrow leaves with a grayish-green color; small pink flowers	Fuzzy textured leaves; it releases a gentle fragrance when the leaves are crushed, adding to the sensory experience	Perennial/shrub/late spring to early summer

### 3.4 Medicinal and edible plants area

**FIGURE 9:**
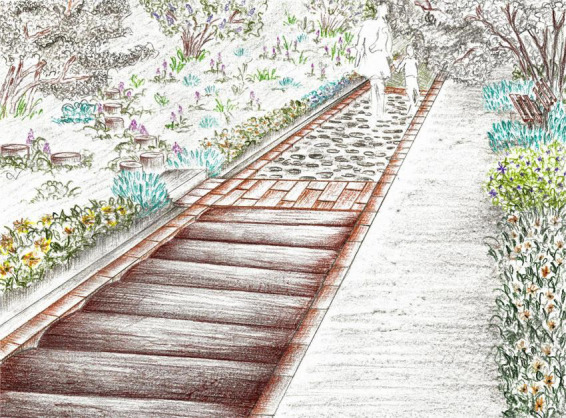
Medicinal and edible plants area view.

This area contains species with curative role and edible plants, supporting the healthcare function of the facility. The sensory alley has a greater variety of materials (mulch, different types of stone, lawn, pine cones, wood, or wood chips) and it is accompanied by a concrete alley on its entire length to facilitate mobility, overall contributing to the principles of a well-organized and accessible space. Besides the generous pathway, the space can also be explored by the adventurous on the scattered stepping wood logs arranged on the slope (Figure 9). Visitors can recognize and learn about the medicinal plants and their healing properties from information panels. Plant harvesting and fruit eating would be suggested activities, encouraging movement and providing gustatory stimulation, being in line with the key findings of providing sensory stimulation and interaction with nature. The proposed plant material that would reflect and enhance the functions of this area, along with various features, are described in Table 2. Various plant species would encourage the presence of wildlife and the dominance of softscape, while the space can be enjoyed also from shaded sitting areas as the literature’s findings suggest.

**TABLE 2:** Plants proposed for the medicinal and edible plants area and their features.

Species	Medicinal/ edible features	Therapeutic effects	Decorative elements	Tactile experience	Plant type/growth habit/decorative period
*Papaver somniferum*	Edible seeds	Medical use for pain relief; attracts birds and other pollinators (Bao and Zhu, 2022)	Large flowers in shades of red, pink, purple or white	Silky petals, smooth leaves	Annual/herbaceous/late spring to early summer
*Helianthus annuus*	Edible seeds	Rich in nutrients seeds; attractive to butterflies, birds, and bees (Pal, 2011)	Large, composite inflorescences with prominent yellow ray florets surrounding a brown disk	Hairy stems, rough leaves	Annual/herbaceous/ summer to early fall
*Matricaria chamomilla*	Flowers	Calming, soothing, reduces inflammation; attracts bees and other pollinators (Mao et al., 2016; Amsterdam et al., 2020)	Small, radiate capitula with white ligulate ray florets and yellow tubular disk florets	Feathery leaves, silky flowers	Annual/herbaceous/ summer
*Pyrus communis*	Edible fruits	Provides vitamins and fibers; attractive to butterflies, birds, and bees (Kolniak-Ostek, 2016; Simionca Mărcăşan et al., 2023)	White blossoms, aesthetically pleasing foliage	Smooth bark and leaves, glossy fruit surface	Perennial/tree/spring to fall
*Amaranthus caudatus*	Edible seeds and young leaves	Rich in nutrients; attractive to butterflies, birds, and bees (Gamel et al., 2004)	Pendulous red inflorescences	Rough leaves and stems, soft inflorescences	Annual/herbaceous/ summer to early fall
*Malus domestica*	Edible fruits	Rich in vitamins and fiber; attractive to butterflies, birds, and bees (Patocka et al., 2020; Sestras and Sestras, 2023)	White or pink flowers in spring, colored fruits in autumn	Rough textured bark, smooth leaves, and fruit	Perennial/tree/spring to fall
*Ribes nigrum*	Edible berries	High in vitamin C, supports immune system health; attractive to butterflies, birds, and bees (Karjalainen et al., 2009)	Racemes of greenish-yellow flowers followed by black berries	Hairy leaves, smooth berries	Perennial/shrub/spring to fall
*Monarda didyma*	Edible flowers and leaves	Antiseptic, antibacterial, antifungal; attractive to butterflies, birds, and bees (Côté et al., 2021)	Vibrant red flowers that attract pollinators	Slightly rough textured leaves, soft flowers	Perennial/herbaceous/ summer
*Rubus idaeus*	Edible berries	High in vitamin C; attractive to butterflies, birds, and bees (Zhbanova, 2019)	White flowers that develop into red berries	Soft and fuzzy leaves; delicate hairy flowers	Perennial/shrub/spring to fall
*Echinacea purpurea*	Roots, leaves and flowers	Boosts immune system, reduces inflammation; attractive to butterflies, birds, and bees (Goel et al., 2005)	Large, daisy-like flowers with purple ligules and orange-brown central cones	Soft and smooth petals	Perennial/shrub/summer to fall
*Centaurea cyanus*	Flowers	Mild anti-inflammatory and soothing properties; attractive to butterflies, and bees (Garbacki et al., 1999)	Blue, purple, pink, or white flowers with involucre bracts	Silky petals, rough leaves	Annual/herbaceous/late spring to early fall
*Achillea millefolium*	Aerial parts of the plant	Anti-inflammatory, antispasmodic, astringent effects; attracts bees and butterflies (Tadić et al., 2017)	Flat-topped corymbs with white, yellow, pink, or red flowers	Feathery leaves, smooth flowers	Perennial/herbaceous/ summer to fall
*Prunus avium*	Edible fruits	Rich in vitamins and antioxidants; attractive to bees, and other pollinators, birds are attracted to the fruit (Kelebek and Selli, 2011; Nunes et al., 2021)	White or pink blossoms in spring, followed by red berries	Smooth bark and leaves	Perennial/tree/spring to fall
*Ribes rubrum*	Edible berries	High in vitamin C, supports immune system health; attractive to bees, and other pollinators, birds are attracted to the fruit (Zdunić et al., 2016)	Greenish-yellow flowers followed by red berries	Slightly hairy leaves, smooth berries	Perennial/shrub/spring to fall
*Calendula officinalis*	Edible flowers	Reduces inflammation, promotes skin health; attractive to bees, and other pollinators (Parente et al., 2012; Silva et al., 2021)	Liguliflorous capitula with bright orange ray florets	Smooth petals, slightly rough leaves	Annual/herbaceous/spring to fall

### 3.5 Occupational therapy area

**FIGURE 10:**
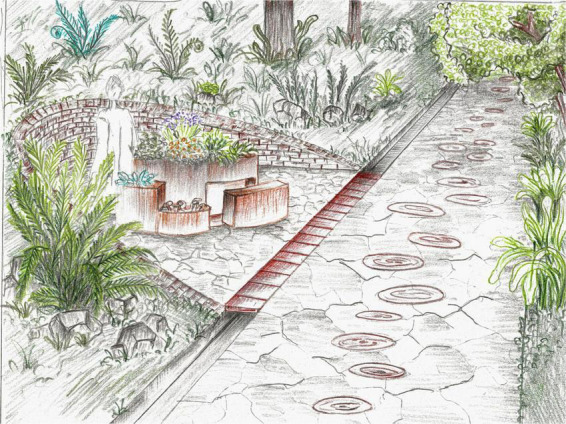
Occupation therapy area view.

Since the importance of activity in therapeutic gardens was highlighted, it was considered necessary to create a space where the patients could be directly involved in gardening activities in specifically designed areas, such as U-shaped pockets in the slopes with accessible raised beds (Figure 10). The area also contains a sand play zone and a water fountain. These features would align with the principles of a well-organized space and encourage interaction with the natural environment. The presence of a water feature (recommended in the key features) associated with indigenous vegetation like the ferns give the place a wilderness vibe, and according to the key feature list, plants would offer sensory stimulation and habitat for wildlife, dominating the hardscape. The space can be explored following a pathway divided into three different sensory-stimulating materials (gravel with stepping stones, grass, stone, and wood slices). The proposed plant material that would reflect and enhance the functions of this area, along with various features, are described in Table 3.

**TABLE 3:** Plants proposed for the occupation therapy area and their features.

Species	Therapeutic effects	Decorative elements	Tactile experience	Plant type/growth habit/decorative period
Ferns	Stress reduction, connection to nature due to its texture; air purification; attracts bees and butterflies (Lee and Shin, 2010)	Delicate, feathery foliage in various nuances of green	Soft, feathery, finely textured fronds offer a delicate tactile experience	Perennial/herbaceous/ spring to fall
*Heuchera* sp.	Raises interest and creates a relaxing environment due to diverse foliage colors (Xu et al., 2023)	Colorful foliage in shade of green, purple, bronze, silver; delicate bell-shaped flowers	Diverse textures from smooth, slightly ruffled, or veined leaves; delicate small flowers	Perennial/herbaceous/ spring to fall
*Juniperus horizontalis*	Calming effects, has evergreen foliage that can create a sense of stability and continuity; provides nesting opportunities for birds (Cantrell et al., 2014)	Attractive blue-green foliage	Needle-like foliage, spiky texture	Perennial/shrub/year-round
*Astilbe arendsii*	Attracts pollinators, promotes relaxation (Kharchenko et al., 2016)	Plumes of flowers in various colors	Feathery flowers	Perennial/herbaceous/ spring to late summer
*Cerastium tomentosum*	Provides visual contrast and rises interest; attractive to bees and other pollinators (Khalaf and Stace, 2001)	Silvery foliage and masses of small star shaped white flowers	Soft, velvet textured leaves	Perennial/herbaceous/ spring to fall
*Primula Veris*	Colorful flowers can uplift moods and reduce stress stimulating the senses; attracts bees and butterflies (Tarapatskyy et al., 2021)	Bright colorful flowers	Smooth flowers with silky texture	Perennial/herbaceous/ spring
*Yucca filamentosa*	Provides visual interest; attracts pollinators (Althoff et al., 2005)	Sword-shaped leaves and creamy-white bell-shaped flowers	Stiff leaves	Perennial/shrub/year-round
*Cotoneaster procumbens*	Calming effects, has evergreen foliage that can create a sense of stability and continuity provides habitat for wildlife (Dmitruk et al., 2022)	White small flowers, red berries	Smooth, glossy textured leaves	Perennial/shrub/year-round
*Lobelia erinus*	Colorful flowers can uplift moods and reduce stress stimulating the senses; attractive to butterflies (Folquitto et al., 2019)	Tubular flowers in shades of blue, violet, pink or white	Smooth leaves, delicate flowers	Annual/herbaceous/late spring to early autumn
*Aquilegia vulgaris*	Graceful nodding flowers that can promote relaxation, offer visual stimulation, and attract pollinators (Hassan et al., 2010)	Various colored flowers	Lacy leaves with soft flowers	Perennial/herbaceous/late spring to early summer

### 3.6 Sound healing area

To complete the idea of a sensory-therapeutic garden it is beneficial for the inclusion of a sound stimulation area. The plants from the *Poaceae* family are representative for the pleasant noises they make when wind blows and are characterized as relaxing. The feeling of calmness is highlighted also by their diaphanous appearance. Additional species that would complement the planting are *Populus tremula*, *Betula pendula*, *Campanula* sp., and also the placement of garden bells. Even though this zone has a primarily purpose relaxing and the enjoyment of sound is made by vegetation, tactile exploration experience is also encouraged, since ornamental grasses are known for their captivating texture. The proposed plant material that would reflect and enhance the functions of this area, along with various features, are described in Table 4. The area provides a variety of sensory stimulation trough the presence of plants and not only, this providing habitat for wildlife and dominance of the softscape while supporting and creating opportunities to interact with the materials and relax in shaded sitting area, these components are in alignment with the key features of therapeutic garden design.

**TABLE 4:** Plants proposed for the sound healing area and their features.

Species	Sound-generating features	Therapeutic effects (He et al., 2022)	Decorative elements	Tactile experience	Plant type/growth habit/decorative period
*Koeleria glauca*	Leaf and inflorescence movement	Calming rustling sounds; the seeds can be foods for birds	Silvery-blue foliage	Fine leaves, delicate seed heads	Perennial/herbaceous/late spring to early summer
*Cortaderia selloana*	Inflorescence and foliage movement	Soothing swaying plumes, rustling leaves; the seeds can be attractive to birds	Tall, elegant inflorescences; green to golden foliage	Feathery texture; slender leaves with sharp edges	Perennial/herbaceous/late summer to early winter
*Hordeum jubatum*	Leaf and inflorescence movement	Calming rustling sounds; the seeds can be attractive to birds	Slender, arching inflorescences; green to golden foliage	Fine, feathery texture	Perennial/herbaceous/late spring to autumn
*Pennisetum alopecuroides*	Inflorescence and foliage movement	Relaxing swaying plumes, rustling leaves; the seeds can be attractive to birds	Bottlebrush-like plumes, arching foliage	Fluffy plumes, soft texture	Perennial/herbaceous/ summer to fall
*Miscanthus sinensis*	Foliage interaction and inflorescence movement	Calming rustling sounds, swaying plumes the seeds can be attractive to birds	Arching foliage with silver/pink plumes	Smooth leaves, feathery inflorescences with a silky touch	Perennial/herbaceous/late summer to winter
*Briza media*	Seed head movement and foliar interaction	Soothing seed head movement; the seeds can be attractive to birds	Delicate seed heads	Fine leaves, delicate seed heads that create a light, airy texture	Perennial/herbaceous/late spring to early summer
*Imperata cylindrica*	Foliage interaction and inflorescence movement	Gentle rustling sounds	Striking red to crimson foliage	Smooth leaves with sharp edges	Perennial/herbaceous/ summer to fall
*Hakonechloa macra*	Foliage interaction and plant sway	Delicate swaying	Cascading, bright green foliage	Smooth, flowing texture	Perennial/herbaceous/ spring to fall
*Populus tremula*	Foliage interaction and plant sway	Calming rustling leaves, swaying branches; attracts bees, provides cover and nesting opportunities for birds	Fluttering leaves	Smooth leaves with fine veins and rough branches	Perennial/tree/spring to fall
*Betula pendula*	Foliage interaction and branch movement	Soothing, whispering leaves, swaying branches; attracts bees and other pollinators, provides cover and nesting opportunities for birds	Pendulous, graceful branches; white bark	Smooth leaves, rough textured bark	Perennial/tree/year-round
*Carex oshimensis*	Foliage movement and plant sway	Gentle rustling sounds	Arching foliage in various shades of green	Smooth, arching leaves	Perennial/herbaceous/year-round

### 3.7 The hypo–Allergenic area

Being in the proximity of the Pneumophtiziology Institute, it is necessary to create a safe space for the patients with respiratory diseases, taking in consideration the facility profile, as the key feature suggest. Pollen is considered to be a major allergenic factor and the use of plants with none or few flowers is recommended (such as ferns, *Buxus* sp., *Euonymus* sp.). Non-flowering plants still contribute to the sensory stimulation, especially tactile and visual, and for providing habitat for wildlife. The space has plenty of shaded sitting areas for rest or to enjoy a pleasant conversation in an outdoor space, aligning with the principles. The planned plant material, together with different characteristics, that would reflect and improve the functions of this area are listed in Table 5.

**TABLE 5:** Plants proposed for the hypo-allergenic area and their features.

Species	Allergenic potential	Therapeutic effects	Decorative elements	Tactile experience	Plant type/growth habit/decorative period
*Ilex aquifolium*	Low (female cultivar- does not produce pollen)	Calming effects, has evergreen foliage that can create a sense of stability and continuity; attracts birds with their berries (Obeso, 1998)	Glossy, dark green leaves; bright red berries that add color during autumn and winter	Glossy, stiff leaves with spiny edges	Perennial/shrub/year-round
Ferns	Low, they do not produce pollen	Stress reduction, connection to nature due to its texture; air purification (Mannan et al., 2008; Lee and Shin, 2010)	Delicate, feathery foliage in various nuances of green	Soft, feathery, finely textured fronds offer a delicate tactile experience	Perennial/herbaceous/ spring to autumn
*Nandina domestica*	Low, produces minimal pollen, it’s not known to cause significant allergy issues	Creates a relaxing environment through visually appealing foliage and wildlife attraction; can attract butterflies and birds	Colorful, lacy foliage that changes color throughout the seasons; produces clusters of white flowers in spring and red berries in autumn	Lacy, compound leaves with slender, smooth leaflets	Perennial/shrub/year-round foliage, white flowers in spring, red berries in autumn
*Skimmia japonica*	Low, female cultivars do not produce pollen	Relaxing effects due to the evergreen foliage, evokes the idea of continuity; wildlife attraction; attracts bees and butterflies (Brndevska and Rizovska Atanasovska, 2012)	Glossy, dark green leaves provide year-round interest; fragrant pale pink flowers in spring and colorful berries in autumn	Smooth, glossy leaves; smooth textured small flowers	Perennial/shrub/year-round foliage, flowers in spring, colorful berries in autumn
*Hosta* sp.	Low, produces minimal pollen, is not known to cause allergy issues	Creates a relaxing atmosphere in shading areas due to the bold, visually appealing foliage; attractive to bees and other pollinators (Suzuki et al., 2002)	Large, bold leaves in various nuances of green; spikes of trumpet-shaped flowers	Diverse textured, smooth, veined foliage with slightly wavy or ruffled margins	Perennial/herbaceous/ foliage -spring to autumn, flowers in summer
*Weigela florida*	Low, produces minimal pollen	Suggests calmness and relaxation due to the flexed branches abundant in flowers; attractive to butterflies and bees (Stawiarz and Wróblewska, 2016)	Arching branches with abundant trumpet-shaped flowers	Smooth leaves; delicate flowers	Perennial/shrub/spring to early summer flowering and foliage until autumn
*Heuchera* sp.	Low, produces minimal pollen	Raises interest and creates a relaxing environment due to diverse foliage colors; attracts pollinators (Andrukh, 2017)	Colorful foliage in shade of green, purple, bronze, silver; delicate bell-shaped flowers	Diverse textures from smooth, slightly ruffled, or veined leaves; delicate small flowers	Perennial/herbaceous/spring to autumn foliage, spring to summer flowers

### 3.8 Sound barrier area

Even though the sound study didn’t reveal any concerning results regarding the noise, once the invading vegetation is removed, it is considered necessary to incorporate the abundant use of trees and bushes with great variety of decorative elements to minimize sounds (i.e., *Photinia* × *fraseri*, *Weigela florida*, *Cornus sanguinea*, *Prunus laurocerasus*). This area would not only decrease the sound coming from passing cars passing but also add an aesthetic part to the garden by having an animating and refreshing effect. Table 6 describes the suggested plant material that would represent and enhance the functions of this space, as well as various ornamental aspects. The plant material would serve as visual stimulation, but the absorption of sound would contribute to the auditory comfort of the area, minimizing the external noise, thus enhancing other pleasant auditory stimulation, as the key features suggest.

**TABLE 6:** Plants proposed for the sound barrier area and their features.

Species	Sound barrier features	Therapeutic effects	Decorative elements	Tactile experience	Plant type/growth habit/decorative period
*Taxus baccata*	Dense, evergreen foliage	Calming effect, creates a sense of privacy; provides nesting opportunities and food for birds (Lavabre and García, 2015)	Dark green foliage; bright red fruits	Needle-like foliage, spiky texture	Perennial/shrub/year-round
*Cornus sanguinea*	Dense branching structure with thick foliage	Visual interest due to colorful stems in winter, can have positive impact on mood; attracts bees, birds (Krüsi and Debussche, 1988)	Intense red stems	Slightly rough textured stems	Perennial/shrub/year-round
*Photinia* × *frasierii*	Evergreen with dense foliage	Provides privacy, attractive red new growth can have good influence on mood; attracts bees and other pollinators	Red new growth	Glossy and slightly leathery leaves	Perennial/shrub/year-round
*Philadelphus coronarius*	Dense branching structure	Fragrant flowers can have a calming effect; attracts bees and other pollinators (Mach and Potter, 2018)	Dense white flowers	Soft, delicate flowers	Perennial/shrub/spring to fall
*Weigela florida*	Dense arching branches with abundant foliage	Dense colorful flowers, can have uplifting effect on mood; attracts bees, butterflies (Stawiarz and Wróblewska, 2016)	Dense pink flowers	Slightly waxy -flowers; slightly rough textured leaves	Perennial/shrub/spring to fall
*Prunus laurocerasus*	Evergreen, dense foliage	Dense foliage creates a sense of closure, privacy; attracts bees and other pollinators, birds might be attracted to the fruits (Mach and Potter, 2017)	Glossy, dark green foliage; small white flowers	Glossy, fine leaves	Perennial/shrub/year-round
*Forsythia suspensa*	Dense, arching branches with abundant foliage	Bright yellow flowers can have positive impact on mood by providing a vibrant visual display; the flowers can be attractive to bees and other pollinators (Mach and Potter, 2017)	Bright yellow flowers	Soft, delicate flowers; slightly rough branches	Perennial/shrub/spring to fall

### 3.9 Dedicated employee area

Taking in account the fact that medical staff need privacy, and considering the site’s structure, the most suited zones dedicated to the healthcare professionals are the ones at the left extremities of the garden. The upper area would serve the personnel from the Pneumophtisiology clinic, while the lower area would serve the specialties in its proximity. The vegetation would not present much diversity in colors nor textures, mostly composed by green shrubs. The suggested benches would have the proper dimensions for users to recline, thus creating a non-triggering, neutral background for relaxation. Table 7 details the suggested features and plant material that would accurately reflect and improve upon the area’s many uses, dominating the hardscape, providing habitat for wildlife and privacy as the design principles suggest.

**TABLE 7:** Plants proposed for the staff dedicated area and their features.

Species	Therapeutic effects	Decorative elements	Tactile experience	Plant type/growth habit/decorative period
*Prunus laurocerasus*	Calming effect, creates a sense of privacy and closure due to the evergreen foliage; attracts bees and other pollinators (Mach and Potter, 2017)	Glossy, dark green foliage; small white flowers	Glossy, fine leaves	Perennial/shrub/year-round
*Hydrangea paniculata*	Visual appealing flowers, in contrast with the background can have invigorating effects on the mood; attractive to bees, butterflies, and other pollinators (Mach and Potter, 2017)	Large flower clusters that can range in color from white to pink	Rough textured leaves; soft and fluffy leaves	Perennial/shrub/late spring to fall
*Deutzia scabra*	Visual appealing dense flowers, in contrast with the background can have invigorating effects on the mood; attracts bees and other pollinators (Mach and Potter, 2017)	Dense, white flowers	Coarse leaves	Perennial/shrub/spring to fall
*Viburnum davidii*	Its textures and color combinations provide visual interest; attractive to bees and other pollinators (Sharifi-Rad et al., 2021)	Textured leaves; Small white flowers	Soft and delicate flowers with silky texture	Perennial/shrub/year-round
*Euonymus japonicus*	Variegated leaves rise visual interest, can have invigorating and uplifting effect on mood; can be attractive to bees and other pollinators (Yucedag et al., 2019)	Green variegated foliage	Glossy and smooth leaves with leathery texture	Perennial/shrub/year-round

## 4 Discussion

Green spaces within the proximities of hospitals offer opportunities for patients to break a monotonous daily indoor routine and to spend time in nature. It is also beneficial for family members and visitors to interact with patients in a comfortable, relaxing environment and for the medical staff to have a peaceful place to retreat during a break. Using program elements like the present study suggests can help to redeem underutilized places in healthcare areas and help to transform them into healing environments (Cordoza et al., 2018; Weerasuriya et al., 2019). This paper adds to this body of knowledge by presenting an integrated approach toward designing sensory-therapeutic gardens. Drawing from a range of literature sources focusing on Post-occupancy Evaluations, rather than conducting a traditional literature review, to gather a set of key principles for therapeutic garden design. However, despite the design principles presented in this study, the design of sensory-therapeutic gardens remains a challenge due to the complexities of users’ needs and specific site conditions, underpinning the need for continuous refinement and adaptation of design principles based on site-specific evaluations (Jiang, 2014; Thaneshwari et al., 2018). Our research included a literature exploration of various articles focusing on Post-occupancy evaluations of healthcare facilities, gathering insights from a spectrum of healthcare contexts, further translating these principles into the specific context of a sensory-therapeutic garden design in Cluj-Napoca, Romania (Polat et al., 2019). Implementation of such initiatives and feedback from the users can bring improvements in the design process and thus lead to better understanding about the requirements of a therapeutic garden (Ivarsson and Grahn, 2010; Uwajeh et al., 2019). Post-occupancy research emphasizes the user’s needs, and highlights the positive and negative aspects of the newly created space. This then contributes to the constant improvement of the therapeutic garden design guidelines, aiding specialists in developing enhanced healing environments (Abbas and Ghazali, 2012; Sidenius et al., 2017).

Healing gardens with therapeutic properties are spaces that assist the recovery process and have both physical and spiritual implications (Hastuti, 2020; Hastuti and Lorica, 2020). Drawing from the user feedback and design key principles extracted from the literature exploration, the defining design features of therapeutic gardens encompass: (1) creating a well-organized space, (2) taking in account the facility’s profile when designing the space, (3) prioritizing softscape over hardscape, (4) supporting human-nature interaction, (5) ensure access and visibility, (6) provide plenty of seating and shaded areas, (7) include water features (8) design secluded areas for staff, (9) use vegetation and natural elements for multi-sensory stimulation, and (10) encourage biodiversity. This comprehensive set of principles provides a blueprint for the sensory-therapeutic garden design, that aligns with the previous findings (Vapaa, 2002; Asano, 2008; Pouya and Demirel, 2015). Furthermore, the design process presented in this study demonstrates how the principles derived from the literature exploration and user feedback were translated into a specific garden design, thus proving their applicability in real-world design scenarios.

The design features of the therapeutic garden should ideally meet the specialization of the hospital and the pathology of the users, as different types of patients have different needs. It is important to emphasize that not all pathologies have been extensively researched in terms of therapeutic gardens design, thus there still might be cases where in order for the user’s needs to be met, the specialists creating the garden need to rely on their professional experience and on a collaboration with the end user in developing the design (Eckerling, 1996). For example, garden design for cancer patients should have different properties than the one designed for children, mentally ill patients, or hospital staff in terms of colors, activities, fragrances, and accessibility; but they all should create a safe space with plenty of vegetation, provide shade, comfortable seating and facilitate independent movement (Fleming and Figueiredo, 2013; Paraskevopoulou and Kamperi, 2018). These are facts that are in concordance with the design proposal, having specific vegetation for lung pathology-related patients, providing medicinal plants and fresh fruits through the medicinal and edible plants area, that enhance the tactile sensory experience for patients with visual impairments.

By design, the aim of sensory-therapeutic gardens is to offer sensory experiences, with a recreational and/or educational outcome, actively engaging its visitors in exploration by stimulating all the senses: tactile, gustatory, olfactory, visual, auditory and movement (Hussein, 2010; Stepansky et al., 2022), the present proposal includes these features by integrating tactile, visual or auditive stimulating vegetation. Such gardens, besides a senses-themed design, should afford activities like fruit harvesting, exploration, interaction with the environment by using species with interesting foliage, incorporating water features, diversifying hardscape materials, stimulating curiosity, being accessible to all visitors, and to encourage social gathering (Hussein et al., 2016; Siu et al., 2020), a requirement sustained by the proposed occupational therapy area.

A very important part of a successful therapeutic design would be the patient’s, family members and medical staff involvement and/or opinion in the design process from the beginning, since they will be the final users of the space, and maybe have different expectations, needs or ideas for the garden (Heath, 2004; Senes et al., 2012). This was an aspect that in this study was not considered. Also, staff members are directly involved in the patient’s recovery and are as equally important when making design choices about their working environment. Research indicates a significant gap in finding what medical staff needs in order to provide quality services while preserving their own health (Huisman et al., 2012; Smidl et al., 2017), so in the present design proposal it was considered necessary to integrate a secluded area for the medical staff. Besides recommendations from scientific literature or user feedback, another factor that might influence the outcome of a therapeutic garden design is the site’s location and surroundings. Aspects like the terrain topography (slopes, flat terrain, or mixed), climate (influences the vegetation palette that can be used in the design), vicinities (can bring the necessity to create sound barriers being located near a noisy area, or some sights might need to be highlighted or, conversely, hidden) can have major implications in the decision making process regarding the zoning, functions and aesthetics of the garden (Salamy, 1995; Shahrad, 2012). In the present study, the fragmented space led to the partitioning of the functional zoning of the space.

While POE method can provide valuable information on designing healing spaces, more modern evaluation methods can be used, that offer more objective, time efficient and standardized approaches. For instance, in individuals with dementia, the positive impact that the garden exposure had on the mood and reduced medication was reflected in the lower levels of cortisol and blood pressure, offering a more accurate evaluation of the therapeutic effects (Pedrinolla et al., 2019; Murroni et al., 2021). Electroencephalogram technology is a non-invasive approach that allows the measurement of mood indicators, and in the context of evaluating individuals participating in gardening-related activities, revealed that, in addition to experiencing relaxation, participants also felt a sense of interest and engagement in the activity (Du et al., 2022). Other methods, like wearable sensors can be used to measure physiological responses to outdoor environments, a method that, combined with the use of virtual reality can reveal the potential impact of a proposed design even before it was built (Skulimowski and Badurowicz, 2017; Zhang et al., 2021). Eye tracking technology can reveal insights on what elements of the garden capture visitors’ attention and for how long, allowing designers to understand what elements are most engaging and appealing, or aspects that may be less successful in capturing interest, and even more, it can help uncover any discrepancies between what the subjects verbally report as their preferences and what their gaze patterns disclose (Amati et al., 2018; Junker and Nollen, 2018).

The evolution of technology and new findings in medicine provide hospitals with equipment that improves the quality of the medical services. However, studies have shown that often this is not enough, and the quality of the environment around the patient has a major impact on the recovery process and length of hospital stay. There has been a preference for the design of old hospitals as compared to the new ones, and there is a correlation between the time spent in the hospital and the user’s satisfaction level concerning the surrounding environment. This shows that over time, designers have lost something of essential value in conceiving healing environments (Ghazali and Abbas, 2012b).

## 5 Conclusion

Since ancient times man has felt a deep connection with nature and natural environment, always seeking to integrate it in the healing process, and green spaces around hospitals can provide a great opportunity to create sensory-therapeutic gardens. This study has shed light on the importance of these types of spaces showcasing their potential to facilitate interaction not only between people, but also to encourage a human-nature connection, to provide access to all visitors, and to create a recreational space also for staff and family members. Not only does this enrich the aesthetic of a place with great variety in color and texture given by the materials and vegetation used, but also can invite for further investigation, to bring curiosity into the visitor’s mind. They can create opportunities for outdoor activities like gardening or sensory exploration of nature, by using intriguing and captivating elements in the design but they can serve also as contemplative spaces where one can just spend time outdoor observing nature in silence. Since there are not any precise guidelines for how sensory-therapeutic garden should look like, the design often reflects the landscape designer’s perspective and intuition, but post-occupancy studies often reveal new perspectives, bringing constant improvements. Through an exploration of scientific literature focusing on Post-occupancy evaluations, we have identified key features of healing landscapes that contribute to the effectiveness of these spaces. This paper offers an innovative perspective by translating these general principles into a specific design context, filling a gap in existing guidelines, bringing practical aspects for future sensory-therapeutic garden design, combining literature-derived principles with user feedback and site-specific condition, demonstrating the flexibility and adaptability of these features. However, it is crucial to acknowledge that further research is needed in order to continually refine and improve de design of healing spaces by integrating emerging technologies, enabling more evidence-based design practices.

## Data availability statement

The original contributions presented in the study are included in the article/supplementary material, further inquiries can be directed to the corresponding author.

## Author contributions

MD and AS: conceptualization, resources, project administration, and funding acquisition. MD, AD, and PS: methodology and software. PS, MB, RB, and AS: validation. DD, PS, and MB: formal analysis. MD, D-MM, and DD: investigation. MD and DD: writing–original draft preparation. PS, RB, and AS: writing–review and editing. MD, D-MM, and PS: visualization. MB, RB, and AS: supervision. All authors have read and agreed to the published version of the manuscript.
